# Impact of different CAD/CAM materials on internal and marginal adaptations and fracture resistance of endocrown restorations with: 3D finite element analysis

**DOI:** 10.1186/s12903-023-03114-8

**Published:** 2023-06-25

**Authors:** Shaimaa Ahmed Abo El-Farag, Fatma Abdallah Elerian, Abdallah Ahmed Elsherbiny, Mahy Hassouna Abbas

**Affiliations:** 1 Fixed Prosthodontics Department, Faculty of Dentistry, Mansoura University and Horus University (HUE), Mansoura, Egypt; 2 Production and Mechanical Design Department, Faculty of Engineering, Mansoura University, Mansoura, 35516 Egypt; 3 Fixed Prosthodontics Department, Faculty of Dentistry, Mansoura University and Delta University for Science and Technology, Mansoura, Egypt

**Keywords:** Endocrown; Molars; PEKK; Prettau Zirconia; Hybrid ceramic; Finite Element Analysis

## Abstract

**Purpose:**

To assess and compare the impact of various computers aided design/manufacturing (CAD/CAM) materials on internal and marginal discrepancies, fracture resistance and failure probability of Endocrown restorations with 3D Finite Element analysis.

**Material and methods:**

Forty devitalized human maxillary first molars were collected. After endodontic treatment, they classified into 4 groups (*n* = 10) based on the materials used for endocrown fabrication. Group V (Vita-Enamic), Group N (Nacera Hybrid), Group T (Translucent Prettau Zirconia) and Group P (Pekkton ivory). All samples were exposed to artificial aging method simulating one year of clinical service. Silicone replica technique and stereomicroscope (25X) utilized to evaluate the marginal and internal gaps of endocrowns at different areas. Fracture resistance test used for cemented specimens followed by qualitative investigation utilizing Stereomicroscopy. Four models representing four endocrown systems used for restoration of severely-damaged endodontically treated upper first molar were generated for finite element analysis (FEA). Axially and centrally static occlusal compressive load was applied. Modified Von Mises and maximum principal stress values on the remaining tooth structure, cement lines and restorative materials were assessed independently. Resulted data were statistically analyzed at *P*-value ≤ 0.05.

**Results:**

In the current study, the highest mean values of internal and marginal discrepancies (μm) among studied groups were reported for Zirconia group (100.300 and 102.650) respectively, while the lowest mean value of internal discrepancy (μm) was observed for Nacera group (69.275) and the lowest mean value of marginal discrepancy (μm) was observed for PEKK group (78.4750). Regarding internal discrepancy, Vita-Enamic and PEKK groups did not exhibit any statistically significant differences (*P* = 0.293), however zirconia and the other tested groups exhibited statistically significant differences in the mean values of the marginal gap region (p 0.05).On the other hand, PEKK group showed the highest mean value of fracture resistance (1845.20 N) and the lowest value was observed for Vita-Enamic group (946.50 N). Regarding to stress distributions through 3D FEA, and according to modified von Mises (mvM) analysis, the greatest possible stress values were noticed in PEKK model in relation to tooth structure, cement line, and flowable composite as the following: (93.1, 64.5, 58.4 MPa) respectively, while Zirconia revealed lower maximum stress values (11.4, 13.6, 11.6 MPa) respectively.

**Conclusions:**

Statistically excellent marginal and internal fit was observed for PEKK in relation to other used endocrown materials. Also, PEKK material explained fracture resistance comparable to zirconia value while the lowest value was detected for Vita Enamic material.

**Supplementary Information:**

The online version contains supplementary material available at 10.1186/s12903-023-03114-8.

## Background

Endodontic treatment is considered typical dental procedure for treatment of badly destructed teeth. It causes structural changes of treated teeth dentin such as loss of the water and collagen content. This can explain why teeth that have had endodontic treatment (ETT) are more susceptible to biomechanical failure than healthy teeth. The restoration of these teeth is considered a clinical challenge, as they have a reduced fracture resistance and stiffness. Instead of dryness or physical changes in the dentin, this decline appears to be linked to the loss of structural continuity caused by trauma, caries, protracted cavity preparation, or other factors [1]. According to biomechanical principles, the structural strength of a tooth structure depends on the hard tissues quantity as well as anatomic form integrity and intrinsic strength [2]. Following endodontic treatment, variations happened in tissue quality, have insignificant effect on biomechanical behavior of tooth. Conservative endodontic access cavity proved to have minimal influence on the tooth fracture resistance from mechanical point of view [3].

Endodontically treated tooth (ETT) restoration has long been a contentious topic. Maintaining the integrity of the residual dental tissue and choosing a practical restorative material for both restoration and the structural strength of the tooth are crucial in the case of such teeth [4]. Teeth with minor coronal tooth structural loss may require adhesive restoration such as composite resin, while complete coverage restoration may be suggested when there is sufficient amount of coronal tooth structure allowing restoration adhesion and stability. More loss of coronal tooth structures with limited ability for adhesion and stability makes the post and core restoration mandatory. In case of severely destructed teeth with most of lost coronal tooth structure, extraction and dental implant may be acceptable line of treatment [2, 5].

Endocrown restorations have been designed as an alternative to post-core systems for the repair of badly destructed teeth as adhesive dentistry and the development of all-ceramic materials with good mechanical qualities have allowed the treatment using the post and core strategy less noticeable [6, 7]. Endocrowns are monoblock restorations that integrate core structure and crown restoration utilizing both the micro-mechanical retention of the adhesive cementation and the macro-retentive support of the pulp chamber walls [8].

Comparing Endocrowns to traditional crowns restored using a cast post and core or a fiber post and resin core, it has been found that Endocrowns are more resistant to fracture. They also have excellent esthetic qualities, require less clinical time, are simple to use, and are inexpensive [9, 10]. They are generally suggested in circumstances when there has been a significant loss of crown tissue, the patient has a lack of interproximal or interocclusal space, and conventional post-and-crown rehabilitation is not feasible due to insufficient ceramic thickness. Additionally, they serve as a replacement for teeth with short or atrophic clinical crowns and root canals that are too short, curved, or calcified for post application [9].

Preparation of Endocrown restorations differs from that of conventional complete crown as being adhesive restoration not require subgingival margin placement with subsequent inflammatory effect on gingival tissue [11, 12]. There are general guidelines of Endocrown restorations such as: 90^◦^ butt margin, 2-3 mm cuspal reduction, internal taper 6–10 degree, and smooth internal transition [13–15]. Utilizing the space inside the pulp chamber increased the restoration’s stability and retention, but it is difficult to estimate the exact size of the central retentive pulp cavity, particularly when there is significant tooth structure loss and just 1–2 mm of preserved tooth structure above the cement-enamel junction. The extent of surface area that is accessible for adhesive retention and masticatory force distribution is clearly impacted by the depth of the pulp cavity and the consequent intracoronal extension [16]. An essential area of interest is the selection of the restorative material to enhance the efficacy of such Endocrown restoration. Recently, CAD/CAM ceramics with higher restorative adaption, increased mechanical characteristics, and superior optical properties have been presented [17, 18]. The strongest dental ceramics, Zirconias, are increasingly created in monolithic form for a variety of clinical uses. The most prevalent type is Y-TZP (yttria-stabilized tetragonal zirconia polycrystalline) [19]. Prettau® Zirconia is the material of choice for frequently occurring problems like reduced available space, bruxism or ceramic chipping. It offers a functional and at the same time esthetical solution. This highly biocompatible and non-porous material distinguishes itself through its extremely high translucency and perfectly natural appearance.

For CAD/CAM (computer aided design and computer aided manufacturing) technology, composite hybrid ceramics have recently been developed with improved physical and mechanical properties by changing their manufacturing processes, through high temperature and/or high-pressure new polymerization mode, and structure (glass ceramic networks) [20]. Clinically, CAD/CAM hybrid ceramics, which are less rigid and hard versus monolithic ceramics, minimize wear to the opposing tooth structure. They are also less fragile than ceramics [21], with less chipping and greater marginal quality materials are easily machined and manufactured [22]. According to their microstructural geometry, CAD/CAM resin hybrid ceramic blocks can be divided into two primary groups: The first type is polymer-infiltrated ceramic networks (PICN), which are high-temperature and high-pressure polymerized and consist of 14% resin embedded in 86% of ceramic network (e.g., Vita-Enamic) [20]. It is based on infiltration of pre-sintered ceramic network (In-Ceram System) which was introduced by Vita in the 90’s [21, 22] with a low-viscosity acrylate polymer network by capillary action [23–25].

The other type is Nacera® Hybrid that is the new CAD/CAM material for chairside or labside milling machines. A new millable Nacera Hybrid material was recently launched to the dental market. The manufacturer claimed that this new material meets all requirements of a modern, multi-functional composite for CAD/CAM technology, combining ceramics and composite characteristics. Nacera Hybrid is already a fully polymerized material and does not need firing. It is characterized with acceptable esthetics, an adequate level of elasticity, universal processability, and versatility. It is a high performance hybrid ceramic that can be used for esthetic long-term temporaries or permanent restorations [22].

Dental ceramics have a number of desirable features, although they are reactive to application and processing errors and still exhibit lower tensile and bending strengths than metals [26, 27]. The use of polymers in dentistry as a substitute to ceramics has grown in recent years. High-performance thermoplastic polymers made of polyetheretherketone (PEEK) and polyetherketoneketone (PEKK) are known as polyaryletherketones (PAEKs), according to its definition [26]. PEKK, a more recent material with excellent biocompatibility, offers superior long-term fatigue qualities and a compressive quality that is 80% higher than that of unreinforced PEEK [28, 29]. These materials are thought of as alternatives to metals and ceramics in the dental field because they exhibit improved stress distribution, reasonable fracture resistance, and shock absorption [30–32]. Because of its light weight and compatibility with various veneering materials, it has been used as an alternative material for frameworks of partial detachable dental prostheses, frameworks of partial and complete fixed dental prostheses, dental implants, and implant abutments [33, 34].

Marginal integrity, internal adaptation, and fracture resistance which are critical for the clinical outcome of any dental restoration present the most significant parameters that affect periodontal status and restoration longevity. Increased marginal and internal discrepancies will cause luting cements to dissolve in the oral environment, reducing the durability and increasing the failure rate of the restorations [35]. For clinical long-term success, the fitting accuracy of CAD/CAM manufactured restorations is essential. There is currently a lack of information regarding the internal adaptation and marginal integrity of Endocrown restorations, as well as the impact of various materials on the fitting of Endocrown restorations. Although several materials can be referred to for prosthetic restoration of ETT, there is limited knowledge about the biomechanical behavior when correlating wide range of materials for restoration of damaged ETT. The investigations often use destructive mechanical testing to evaluate how teeth respond when subjected to severe loading. However, a non-destructive technique called “Finite Element Analysis (FEA)” has gained widespread acceptance as an important research tool for analysis of internal structural performance in order to identify long-term failures in particular regions and supplement in-vitro testing.. Being able to run numerous simulations without the need for patients or performing human tests, FEM analysis significantly adds to the development of new technologies and new materials in the biomedical field [36]. It provides researchers to evaluate the biomechanical characteristics of dental restoratives, prosthodontics materials and supporting oral tissues that are challenging to be measured clinically. By reducing partial differential equations to a set of algebraic equations, the finite element analysis (FEA) or finite element method (FEM) is a numerical technique for locating approximate mechanical characteristics, also, it provides numerical simulation of the effect of various materials, techniques, and designs regarding the distribution of stress and displacement under specific loads. The primary function of the finite element technique is discretization, which is accomplished by constructing a grid (mesh) from primitives (finite elements) in the coded form (triangles and quadrilaterals for 2D domains, tetrahedra and hexahedron for 3D domains) [37].

The present research work was conducted to determine and compare the impact of different computer-aided design/manufacturing (CAD/CAM) materials on the marginal integrity, internal adaptation, fracture resistance, and failure probability of endodontically treated maxillary molars restored with endocrown restorations using 3D Finite Element Examination.The null hyposthesis was assumed that there are no effect on marginal integrity, and fracture resistance of endocrown restoration with 3D Finite Element analysis using of different CAD/CAM materials.

## Materials and methods

The materials used in this research are described in (Table 1).

**Table 1:** Materials used in this study

Material	Product name	Composition	Manufacturer	Batch number
1) Translucent Prettau Zirconia	Ceramill Zolid HT + White ZrO**2**	partially stabilized with yttrium and enriched with aluminium	(Ceramill Zolid HT, Amman Girrbach, Germany)	1,909,001
2) Vita Enamic	Polymer infiltrated ceramics VITA ENAMIC blocks 2M2-HT-EM-14	Polymer infiltrated ceramic, SiO**2** (58–63), Al**2**O**3** (20–23), Na**2**O (9–11), K**2**O (4–6), B**2**O**3** (0.5–2), ZrO**2** (< 1), KaO (< 1)	VITA Zahnfabrik, Spitaglasses 3, D-79713 Bad Säckingen, Germany	45,810
3) Nacera Hybrid	Tough, fully polymerized radiopaque composite material with optimized, high-density filler technology (Hybrid A2, Block S)	50% Nano-Glass and 50% Polymer-Matrix	DOCERAM Medical Ceramics GmbH Hesslingsweg 65—67 | D-44309 Dortmund / Germany	100,238
4) High performance polymer PEKK	PEKKTON ivory milling blank (98.5/t20mm)	-Polyetherketoneketone ( PEKK) 90% -Titanium Dioxide (TiO**2**) 10%	Cendres + Metaux SA, Biel/Bienne, Switzerland	0000347597
5) Nexcomp	Nano-hybrid composite resin	Bis-GMA, UDMA, Bis-EMA Borosilicate glass	META® BIOMED, Korea	NXC 1,712,112
6) SuperCem, Self-Etch Self-Adhesive Resin Cement	Dual cured dental resin cement, base and catalyst with a dual syringe and mixing tip	**Base:** Silicon dioxide, Barium glass, BisGMA, Triethyleneglycol Dimethacrylate, Diurethan-dimethacrylate **Catalyst:** Silicon dioxide, Barium glass, Tri-ethyleneglycol Dimethacrylate, Diurethan- di methacrylate, Champhorquione	DentKist, Inc, Eli-Dent group S.P.A. KOREA	3,020,004

### Selection and standardization of teeth

The study was led at faculty of Dentistry, Mansoura University, Egypt after approval of ethics committee with number A08041022. After receiving patient consent from the Oral and Maxillofacial Surgery Department, Faculty of Dentistry, Mansoura University, forty healthy human maxillary first molars were collected with properly developed roots that had recently been extracted for periodontal factors from patients requiring complete dentures or diabetic patients. Teeth were movable, hopeless, and periodontal damaged. The teeth were chosen for their uniform morphology and size [34, 38]. At the cement-enamel junction (CEJ), selected teeth were measured with a digital caliper, the average bucco-palatal and mesio-distal dimension widths were 10.73 ± 0.64 mm and 9.31 ± 0.52 mm respectively, with a maximum variance of 10%. Teeth with cracks, caries, or restorations were not accepted. In this study, 5.25% sodium hypochlorite household bleach diluted 1:10 was used to disinfect all chosen teeth for one week at room temperature. Throughout all testing periods, the teeth were kept in distilled water to prevent dryness [30].

### Endodontic treatment of teeth

The same clinician performed endodontic treatments for each of the chosen teeth using a NiTi rotary files system (Race/25 mm) in accordance with the manufacturer's recommendations [1]. Canals were irrigated using 5.25% sodium hypochlorite liquid and the smear layer was finally removed using 17% EDTA solution applied for 5 min. Root canals were dried and sealed with guttapercha points (Meta; Korea) and resin-based root canal sealant after being obturated to their full working length using the lateral compaction technique. (ADSEAL, MetaBiomed, Korea). The excess of gutta percha was removed with a red hot condenser and periapical x-ray was taken [1, 39].

### Teeth mounting

During construction in epoxy resin blocks, a centralizing device (Ahmed’s EL-Din Customized Device-Fixed prosthodontics Department, Faculty of dentistry, Mansoura University) [40] was utilized to enable proper centralization of the teeth. Each tooth was fixed in an upright position with its long axis parallel to the center of the plastic ring (in lower part of device). The occlusal surface of the tooth was adhered to the pin holder of device using a pink wax and centralized in a way that the margin of the epoxy resin (KEMAPOXY 150, CMB chemicals, Egypt) is below the CEJ by 2 mm to simulate the normal biological width. The forty teeth were mounted individually and left for 24 h to gain its maximum hardness, then the plastic ring was removed and blocks were inspected for any defects. Using the Transitional Wax Technique as well as a light-body of polyvinyl siloxane impression material (Harmony light fast setting), a homogeneous 0.3 mm layer of periodontal ligament (PDL) was constructed around the roots of all teeth [41].

### Teeth preparation

After root canal treatment of all the teeth, the guttapercha was removed till canals entrance using a round bur with a water cooling system. The access cavities and floor of pulp chamber cavity were coated using 2 mm thickness flowable composite resin material (Nexcomp shade A2, META® BIOMED, Korea) that used to seal the canal entrance. A thin layer of a light-cured universal dental adhesive (All-Bond Universal) was used before applying composite to optimize bonding [34]. According to the manufacturer guidelines, this adhesive was applied to the cavity for 10–15 s, air-thinned for 10 s, and then light-cured for 10 s using an LED light-curing device (Elipar DeepCure-S). A standardized preparation including all selected teeth was achieved by using of Computerized Numerical Control milling machine (CNC) (C.N.C Premium 4820, imes-icore, Eiterfeld, Germany). It was used to remove the occlusal surface of all teeth horizontally leaving 3 mm above the CEJ from their proximal surfaces using a super coarse diamond disc. The preparation of endodontically treated teeth (ETT) was completed by CNC machine to prepare teeth according to its own preparation criteria with a retention pulp chamber cavity of 8 degrees divergence of the walls [11], butt joint marginal design, 4 mm pulp cavity extension depth which measured from coronal tooth structure to the flowable composite on pulpal floor and circular axial wall thickness of 2 ± 0.5 mm, all internal line angles were rounded and smoothened [34]. Digital caliper was used to confirm the dimensions of prepared teeth for verification. According to the materials used to construct the endocrown, all prepared teeth were categorized into four groups (*n* = 10) as: Group T (Translucent Prettau Zirconia), Group V (Vita-Enamic), Group N (Nacera Hybrid) and Group P (Pekkton ivory).

### Fabrication of endocrown restoration

Ceramill motion 2(5x) (Amann Girrbach, Germany) CAD/CAM system was used for fabrication of 40 endocrown restorations. Four types of CAD/CAM materials such as Translucent Prettau Zirconia (Ceramill Zolid HT, Amman Girrbach, Germany), polymer infiltrated ceramics VITA ENAMIC blocks 2M2-HT-EM-14, (VITA-Zahnfabrik, Bad Säckingen, Germany), Nacera hybrid (DOCERAM Medical Ceramics GmbH, Germany), and high performance polymer PEKK (PEKKTON ivory milling blank 98.5/t20mm, Switzerland) were used for milling of endocrown restorations. The steps for fabrication were performed according to manufacturer’s recommendations as the following: the prepared teeth within their epoxy resin blocks were secured on the scanning tray then scanned by Amann Girrbach scanner (Ceramill Map 400 scanner) to obtain an optical impression. Siladent anti-reflection scan powder (Siladent-Germany: Dental Lab. Materials) was used to get optimal scan to some pulpal extension depth. More images of the prepared teeth within their epoxy resin blocks were captured along the long axis of prepared teeth and from different angles around them. After that these images were computed together to form the final image. The digital photo of the impression appeared then converted into animated photo.

CAD/CAM software (Ceramill Mind, Amann Girrbach) was used for designing the endocrown restoration. The software produced virtual models from the scanned pictures and the automatic margin finder was used for detecting the preparation margin and path of insertion. The scanned specimens were correlated to designed endocrown restoration. To standardize the endocrown design with a 50 µm cement spacing with its distance from this margin (1 mm), each endocrown was planned to have identical occlusal morphology with the same occlusogingival length [11, 34]. One maxillary first molar model from the software library was selected as the main reference model (alternative model selected), and it was then applied and automatically modified for all processed teeth in order to standardize the endocrown morphology for all prepared teeth. The final master model’s exterior measurements of the first tooth were evaluated, recorded, and then accurately used with all subsequent teeth for more precision.After designing of each endocrown, the information was saved in the standard tessellation language (STL) data files, then sent to the milling unit for the milling process. The milling procedure was performed using a computered controlled milling unit Ceramill motion 2(5X). The four types of material blocks were fixed into its place in the milling chamber then the order was given to the milling machine. The milling process run fully automated without any interference. Ten endocrown restorations were dry-milled for Group P utilizing a single Pekkton ivory milling blank as well as sharp, single-bladed, slide-coated milling equipment (CORiTEC). All endocrown restorations have been cleaned for 3 min using an ultrasonic cleaner. Finally, all restorations were secured to the appropriate teeth and checked for adaptation using a sharp explorer and indicator spray (Renfert Occlutec Spray) under magnification loupe 3.5x (Galilean loupe, Gain Express, China) in order to optimize the fit.

### Internal and marginal gaps (µm) measurement

In order to assess the internal and marginal gaps of endocrown restorations in the four examined groups, the silicone replica technique (SRT) was utilized in conjunction with the light-body vinyl polysiloxane impression material (VPS) (Imflex, Metabiomed, Korea) [42, 43]. Each endocrown has been filled with an orange light-body vinyl Polysiloxane impression material (VPS) and has been held in place along the matching tooth’s long axis for five minutes (the light-body material’s setting period) under finger pressure. An interior surface of the tooth was covered with a layer of the light-body after each endocrown had been removed from its corresponding tooth. The orange light-body was stabilized by using a customized plastic syringe into which the purple heavy-body vinyl Polysiloxane impression material (Imflex, Metabiomed, Korea) was injected and also into the tooth to bond and establish the light-body material. The tooth was put inside the syringe until the material get setting, and then the tooth was removed from the syringe leaving the light-body replica adhered to the heavy-body impression material. Each replica was cut from the center in bucco-palatal and mesio-distal directions into four slices named (MB, MP, DB, DP) using a sharp surgical blade no.11 (HuaiAn TianDa Medical Instruments Co, Ltd, China). Each specimen was divided into slices with parallel walls so that they could be seen perpendicularly on the stereomicroscope stage. A digital stereomicroscope (Olympus Model SZ2-ILST, Japan) with an associated digital camera and software (IS Capture) was used to assess the discrepancy between the endocrown and the tooth that represented by an orange-colored light layer at a magnifying power of 25X. Each slice was separated into three areas for easier comparison named as: Pulpal floor, Pulpal wall, and Marginal area. Each replica includes 36 measurements since there are three readings for each area and nine readings for each slice.

### Cementation of endocrown restoration

All endocrowns were treated before cementation according to manufactures’ recommendations as the followings: Zirconia endocrowns fitting surfaces were sandblasted using 50 µm Al_2_O_3_ particles. For both Vita-Enamic and Nacera-hybrid endocrowns, bonding surfaces were etched by using brush with 8% hydrofluoric acid gel (Porcelain etch, DentoBond Porcelain Fix Itena Products, France) for 20 s. then endocrowns were washed with running water for 20 s and dried with moisture-free compressed air for 30 s. Porcelain Silane (DentoBond Porcelain Fix Itena Products, France) was applied into the endocrowns etched surfaces by using brush, left for 60 s till dry. Initially, the bonding surfaces of the PEKK endocrowns were sandblasted with unrecycled 110 µm aluminium oxide (Zeta Sand) at 2 bar (0.2 MPa) pressure for 5 s at a distance of 1 mm and at a 45° angle. Next, properly clean with steam and dry for 20 s with oil-free air [32]. All 40 prepared teeth were etched for 15 s using a 37% phosphoric acid etching gel (N-Etch Etching Gel), thoroughly washed with water, and then gently dried by air. Dental adhesive resin cement (SuperCem, Self-Etch Self-Adhesive) was applied, mixing and application of the luting cement was in accordance with the manufacturer’s instructions. It was blended, applied to the restoration's fitting surface then fully seated onto its corresponding tooth. The excess cement was removed with a brush prior to spot curing. For standardized equal pressure during the cementation, a load of 1 kg was used over cemented specimens resulted in standardized uniform cement film thickness. The bonding assembly was light polymerized for 40 s for each surface from four directions at a distance of 10 mm. After excess cement has been removed, the constant load was left for 5 min [44].

### Thermal-cycling, fracture testing and failure analysis

All specimens were artificially aged for 24 h after cementation and then preserved in distilled water at 37 °C in an incubator to mimic intra-oral environmental conditions [1, 45]. All specimens were put through 10,000 cycles of temperature changes between 5 °C and 55 °C with a dwell time of 30 s in each distilled water bath and a transfer time of 5 s using a thermal-cycling simulation machine (Thermocycler, Robota, Alexandria, Egypt), simulating nearly one year of clinical service [46, 47]. All specimens were conducted through a fracture strength test utilizing a Universal Testing Machine (Model 3345; Instron Industrial Products, Norwood, MA, USA) with a load cell of 5KN then, computer software (Instron® BluehillLite Software) was used for recording data. Each cemented endocrown was loaded and locked into the testing device’s lower fixed compartment individually. Until permanent deformation or failure, the compressive load was delivered axially and centrally with a load cell of 5kN force using a 6 mm diameter, stainless steel ball-shaped loading piston at a cross head speed of 0.5 mm/min [1, 41]. The highest load-to-failure value was measured in Newtons (N), and mean values for each group were calculated. Under a stereomicroscope with a 25X magnification, the failure mode was identified and classified as either favorable (repairable) or unfavorable (not repairable) based on a 2-examiner agreement [8]. When the failure above CEJ and the cause of failure was only de-bonding and/or cohesive fracture of the restoration or within endocrown, it was considered as favorable fracture. On the other hand, the failure was considered unfavorable if the tooth fracture was below the CEJ including vertical root fracture.

### Finite element analysis in three dimensions (3D FEA)

This technique was employed in this study to assess the internal structural behavior and stress distribution in all endocrown materials, cement lines, and the remaining tooth structure (enamel and dentin), with the application of an axial force [1]. The subsequent work has been done as the following:

- *Creation of finite element models*

Three dimensional geometry of prepared maxillary molar was obtained by scanning technology. The clean, dry, prepared molar was scanned using a highly sensitive 3D optical scanner (Identical Hybrid Scanner, Medit Corp, Seoul, Korea) with a blue LED light source and triple camera scanning technology. CAD 3D modeling software (SOLIDWORKS® 3D CAD, Dassault Systems, Ile-de-France, France) was used to create a 3D solid model of the tooth and endocrowns using the scanning data that were stored as STL files [6, 32]. The bone and periodontal ligament’s geometry for supporting teeth was designed. Around the root, a homogeneous 0.3 mm layer of PDL emulation was created [17]. Furthermore, a 3D epoxy resin cylindrical block for simulating bone was created and endocrown preparation was designed as in-vitro study with cement space of 50 µm.

- *Mesh creation*

**Fig. 1:**
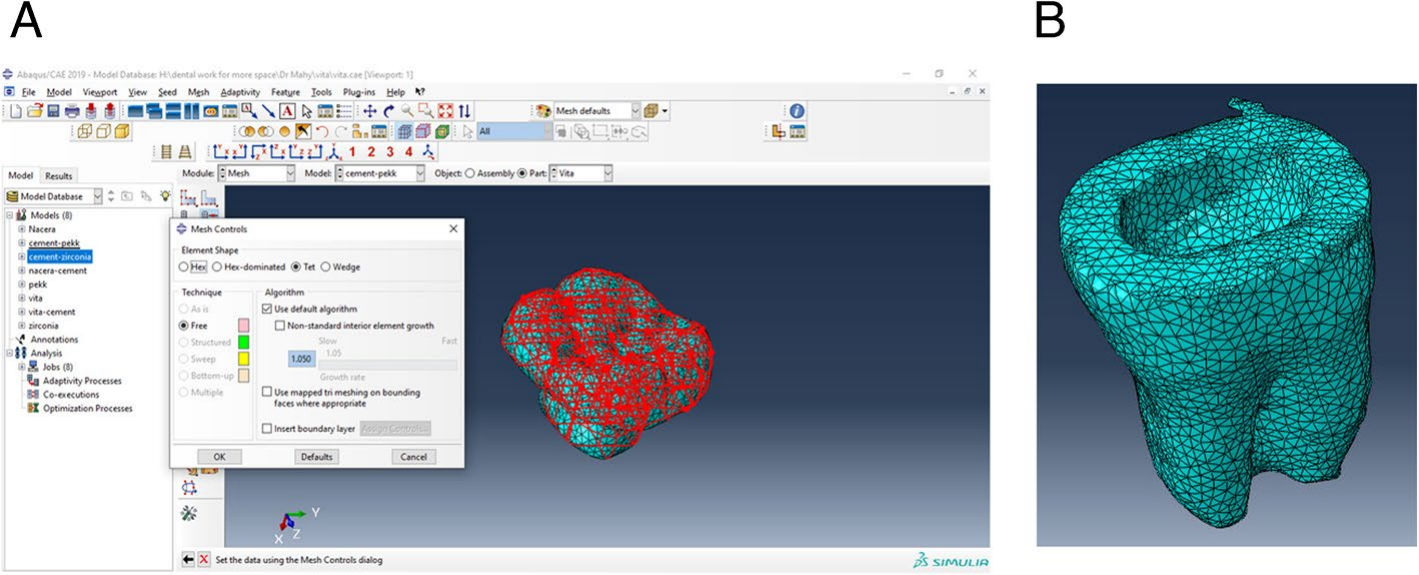
Showing **A **Mesh design and **B **Mesh tooth

The finite element mesh was created and revised using FEA software (Abaqus, 3DEXPERIENCE R2019x®, Dassault Systemes Simulia Corp, Providence, RI, USA) after the geometric 3D solid models for all endocrown materials were produced [10, 48]. Linear tetrahedral elements type C3D4 was used [1, 17, 36]. There were about 26,128 elements and 40,516 nodes in endovrown model and about 52,004 elements and 76,455 nodes in tooth model (Fig. 1). In this software, definition of tooth structure and length of the root, restoration of the cancellous bone to 0.7 mm and periodontal ligament space to 0.3 mm were positioned around the teeth and determination of the cement space of 50 µm was performed.

- *Material data*

Based on the literatures [27, 48, 49] and the manufacturer, mechanical characteristics of the dental structures that were simulated (dental tissues and materials), including Young’s modulus and Poisson’s ratio, were determined and described in (Table 2). Young’s Modulus measures the stiffness of an elastic material, whereas Poisson’s ratio measures the proportion of axial strain (in the direction of the applied load) to transverse strain (perpendicular to the applied load) [27]. For simplicity and to overcome computational challenges, all interfaces were taken into account to be fully bonded, and the model structures were assumed to be linearly elastic, isotropic, and homogeneous [1].

- *Model fixation and loading*

**Table 2:** Mechanical properties and Weibull moduli of the finite element models’ utilized structures

	**Young’s modulus (MPa)**	**Elastic modulus (GPa)**	**Poisson ratio (V)**	**Characteristic strength (MPa)**	**Weibull modulus (*m*)**
Vita Enamic	37,800	x	0.24	193.45	18.80
Zirconia	x	206.3	0.25	700	x
Nacera hybrid	9900	x	x	490	x
PEKK	5100	-	0.40	215	200
Spongious bone	1370	1.37	0.3	x	x
Cortical bone	10,700	x	0.3	x	x
Enamel	84,100	84.10	0.33	42.41	5.53
Dentine	18,600	18.60	0.32	44.45	3.35
Pulp	x	0.0068	0.45	x	x
Periodontal ligament	68.9	0.07	0.45	x	x
Gutta percha	0.69	0.07	0.45	x	x

X: Unavailable value through literature or manufacturer

**Fig. 2:**
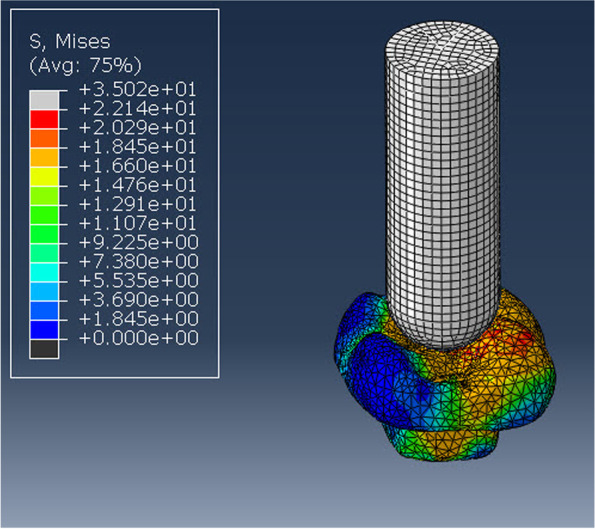
Showing axial and central load application with spherical solid rigid material (SRM)

After establishing the mechanical characteristics of the materials, boundary conditions, loading angle, and element arrangement, software analysis was conducted. A static compressive load applied axially and centrally using a load cell with a 5 k N force was used to complete the analysis. Until the model failed, a crosshead speed of 0.5 mm/min was applied using a 6 mm-diameter spherical solid rigid material (SSRM). A structural linear static analysis was carried out to evaluate the distribution of stress over critical region (Fig. 2). The suitable stress representation measure was detected according to the assessment of failure predictive potential of the conducted analysis. The equivalent stresses (von Mises stresses) energetic criterion was then considered to be more representative of multiaxial stress state. Modified von Mises (mvM) on the molar tooth, cement layer, and restorative ceramic materials were analyzed in Megapascals (MPa) as a distinct component to study stress distribution and position for all endocrowns. The findings are shown as a linear colour scale, with blue denoting the lowest stress levels and red and light grey denoting the highest values for all models' stress distribution.

## Results

The obtained results were subjected to statistical analysis by SAS computer program (Version 9.1.3. SAS Inst., Cary, NC.) using the general linear models (GLM). Data were presented as mean ± SD. One-way analysis of variance was used to compare test groups (ANOVA) and significance of the mean difference between the groups were done by Tukey’s multiple comparison test at (*p* ≤ 0.05). The descriptive statistics including the mean, standard deviation, minimum and maximum values of the internal adaptation and marginal gaps (µm) among the studied groups with different surfaces and regions are shown in (Tables 3 and 4).

**Table 3:** Descriptive statistics of internal and marginal discrepancies (μm) among studied groups

**Internal discrepancy**			**Bias**	**Std. Error**	**95% Confidence Interval**	
					**Lower**	**Upper**
Vita Enamic	N	10				
	Mean	83.1500	-.0371	1.6620	80.1282	86.7000
	Std. Deviation	5.75447	-.58330	1.63998	2.21020	8.03690
Nacera	N	10				
	Mean	69.2750	.0059	1.0570	67.2013	71.3750
	Std. Deviation	3.55209	-.23894	.60208	2.04549	4.33842
Zirconia	N	10				
	Mean	100.3000	.0758	1.1965	98.1256	102.8744
	Std. Deviation	3.97876	-.22735	.62354	2.36117	4.89645
PEKK	N	10				
	Mean	80.5625	-.0058	1.0826	78.3125	82.5625
	Std. Deviation	3.63779	-.20774	.62834	1.98191	4.55598
**Marginal discrepancy**		**Mean**	**Std. Deviation**	**Std. Error**	**95% Confidence Interval for Mean**	
	**N**				**Lower Bound**	**Upper Bound**
Vita Enamic	10	86.5500	14.09777	4.45811	76.4651	96.6349
Nacera	10	82.3750	5.84671	1.84889	78.1925	86.5575
Zirconia	10	102.6500	4.00729	1.26721	99.7834	105.5166
PEKK	10	78.4750	5.15651	1.63063	74.7863	82.1637

**Table 4:** Comparison of internal and marginal discrepancies (μm) between different groups

**Internal discrepancy**		**Mean**	**Std. Deviation**	**Std. Error Mean**	**95% Confidence Interval of the Difference**		***P*-Value**
					**Lower**	**Upper**	
Pair 1	Vita—Nacera	13.87500	8.48712	2.68386	7.80368	19.94632	0.001
Pair 2	Nacera—Zirconia	-31.02500	5.10508	1.61437	-34.67695	-27.37305	0.000
Pair 3	Zirconia—PEKK	19.73750	5.61757	1.77643	15.71893	23.75607	0.000
Pair 4	Vita—PEKK	2.58750	7.32719	2.31706	-2.65405	7.82905	0.293
Pair 5	Vita—Zirconia	-17.15000	7.55370	2.38869	-22.55359	-11.74641	0.000
Pair 6	Nacera—PEKK	-11.28750	4.19327	1.32603	-14.28718	-8.28782	0.000
**Marginal discrepancy**		**Mean**	**Std. Deviation**	**Std. Error Mean**	**95% Confidence Interval of the Difference**		***P*-Value**
					**Lower**	**Upper**	
Pair 1	Vita – Zirconia	-16.10000	15.68403	4.95973	-27.31968	-4.88032	0.010
Pair 2	Vita—PEKK	8.07500	14.43282	4.56406	-2.24962	18.39962	0.111
Pair 3	Vita—Nacera	4.17500	11.54704	3.65149	-4.08525	12.43525	0.282
Pair 4	Zirconia—PEKK	24.17500	4.45666	1.40932	20.98690	27.36310	0.000
Pair 5	Zirconia—Nacera	20.27500	7.98823	2.52610	14.56057	25.98943	0.000
Pair 6	PEKK—Nacera	-3.90000	8.54010	2.70062	-10.00922	2.20922	0.183

**Fig. 3:**
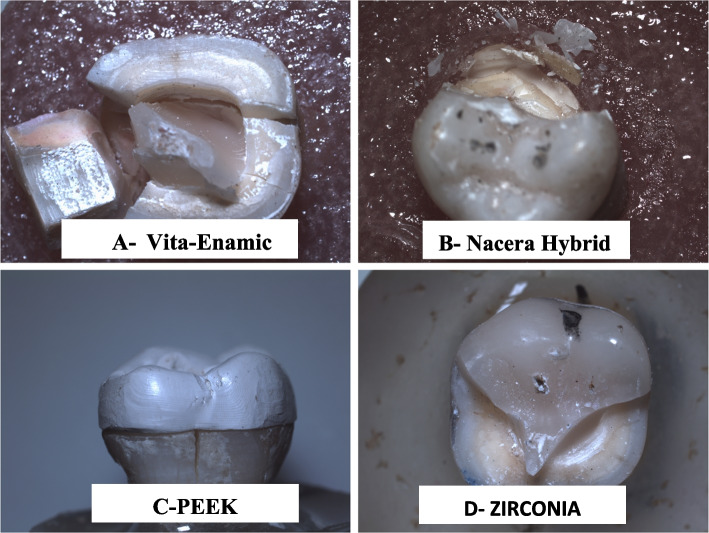
Showing failure modes of all tested materials: **A**, **B**, and **C** unrepairable fracture. **D** is repairable fracture

In the current study it was found that, the highest mean values of internal and marginal discrepancies (μm) among studied groups were reported for Zirconia group (100.300 and 102.650) respectively, while the lowest mean value of internal discrepancy (μm) was observed for Nacera group (69.275) and the lowest mean value of marginal discrepancy (μm) was observed for PEKK group (78.4750). Regarding to internal discrepancy, there was no statistically significant difference between Vita-Enamic and PEKK groups (83.1500, 80.5625 μm) respectively (*P* = 0.293). On the other hand there was statistically significant difference between other groups (*p* = 0.0001). With regard to the results of marginal discrepancy, Vita-Enamic group showed no statistically significant difference with both Nacera (*P* = 0.282) and PEEK (*P* = 0.111) groups, also there was no statistically significant difference between Nacera and PEEK (*P* = 0.183). In addition to previously mentioned results, statistically significant difference was observed between Zirconia material and other tested groups (*p* ≤ 0.05). The PEKK group had the greatest mean value of fracture resistance during the fracture resistance test. (1845.20 N) and the lowest value was observed for Vita-Enamic group (946.50 N), also all tested groups showed statistically significant difference between each other (*p* = 0.0000) as shown in (Table 5). The aforementioned stereomicroscope 25X magnification was used to analyses the broken specimens qualitatively. All specimens were evaluated based on the agreement of two examiners, and the most common mode of failure of all tested groups was shown as percentage within (Table 6), (Fig. 3).

**Table 5:** Mean fracture resistance values in Newton (N) and standard deviations for test groups

One-Sample Test						
	**Test Value = 0**					
	**t**	**df**	**Sig. (2-tailed)**	**Mean ± SD**		**95% Confidence Interval of the Difference**
						**Lower**
**Vita-Enamic**	67.453	9	.000	946.50 ± 44.37		914.7576
**Nacera hybrid**	24.118	9	.000	1135.80 ± 148.92		1029.2682
**Zirconia**	12.301	9	.000		1367.20 ± 351.47	1115.7707
**PEKK**	17.944	9	.000	1845.20 ± 325.18		1612.5784

**Table 6:** Failure modes classification

	**Type I**	**Type II**		**Type III**	**Type IV**		**Type V**	
	**Adhesive failure**	**Cohesive failure**		**Cohesive-Adhesive** **failure**	**Complex fracture** **above the CEJ**	**Total %**	**Complex fracture** **below the CEJ**	**Total %**
	**Non-catastrophic/repairable/ favorable**						**Catastrophic/ unrepairable** **/ unfavorable**	
**VitaEnamic**	0		0	3	4	70%	3	30%
**Nacera hybrid**	0		0	3	3	60%	4	40%
**Zirconia**	0		0	3	1	40%	6	60%
**PEKK**	0		0	2	1	30%	7	70%

### 3D FEA stress distributions

**Fig. 4:**
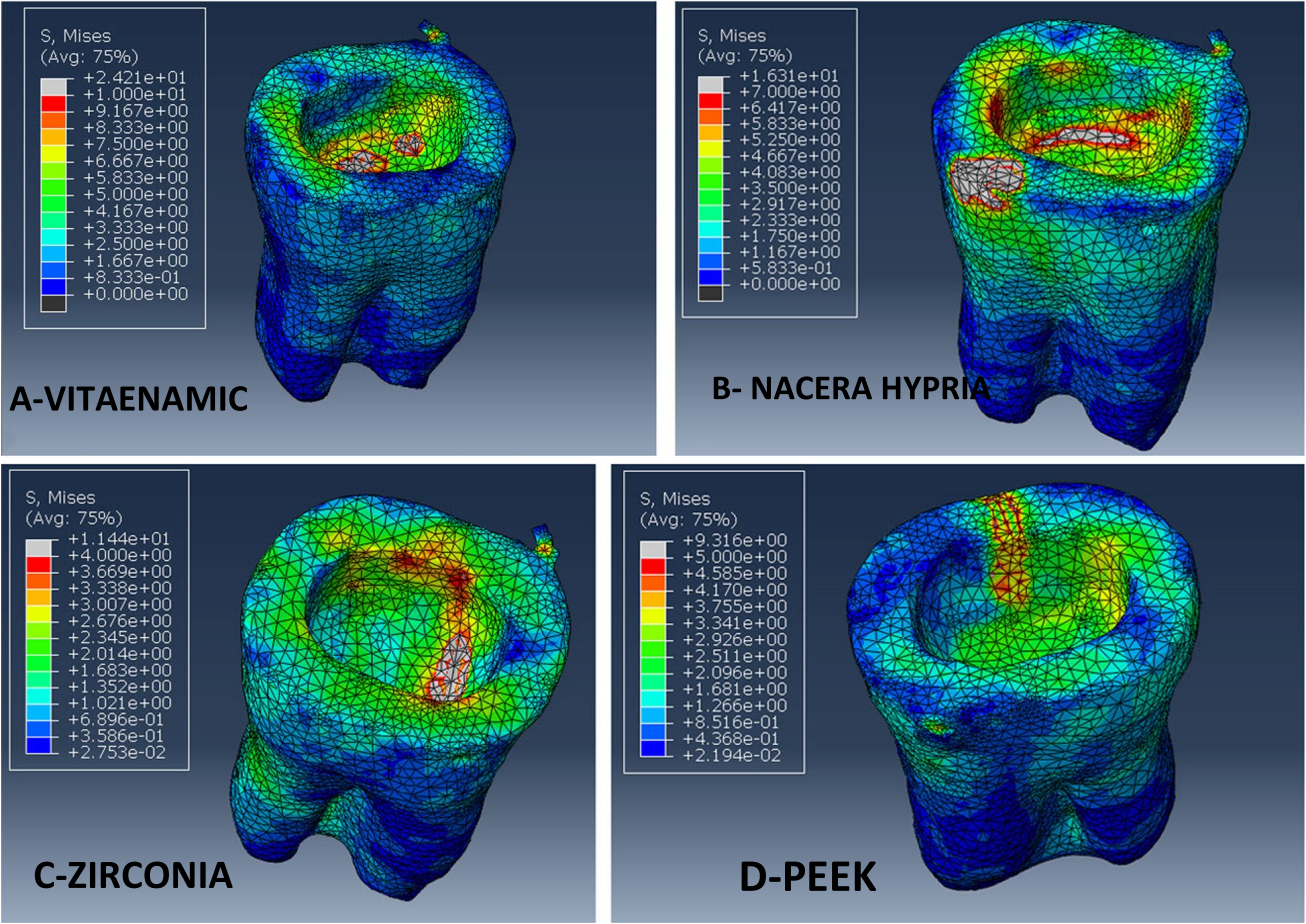
Showing mvM stress distribution pattern for the four models’ tooth structures

**Fig. 5:**
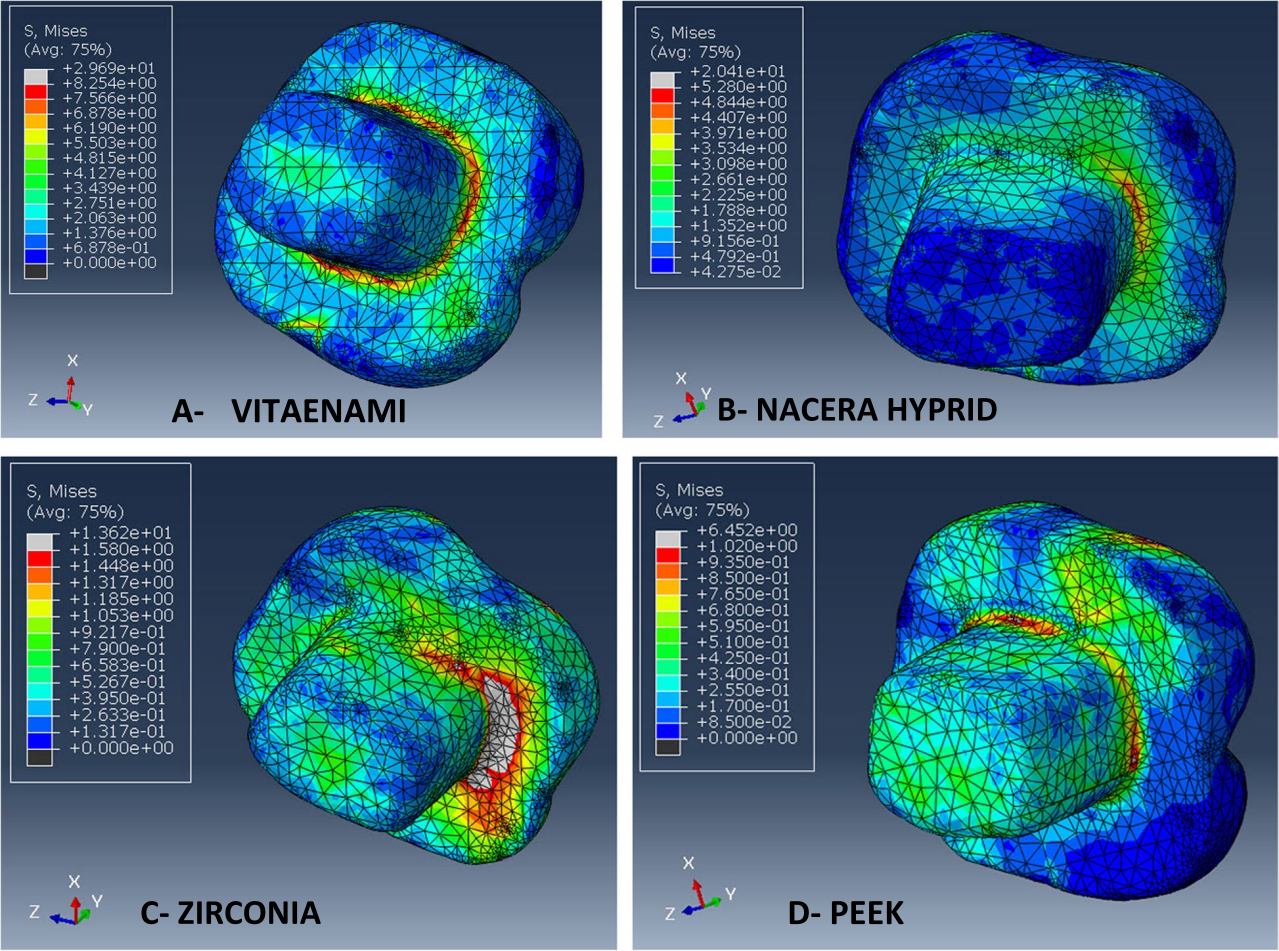
Showing mvM stress distribution pattern for cement line of four models

**Fig. 6:**
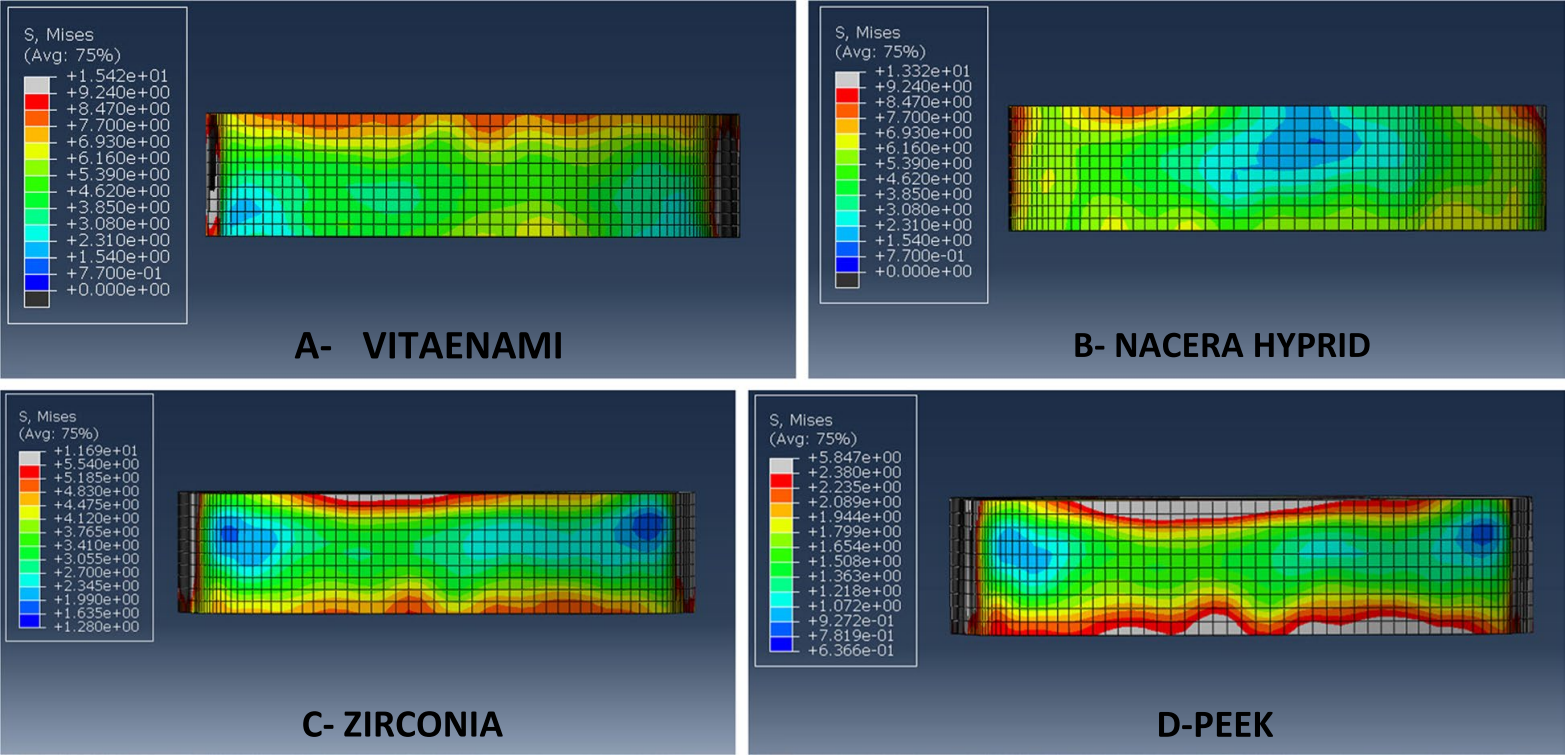
Showing mvM stress distribution pattern for flowable composite of four models

For all evaluated restorative materials, the maximum modified von Mises stress (mvM) values of individual teeth, cement lines, and flowable composite are shown in (Table 7). The highest maximum stress values for the modified von Mises (mvM) analysis were found in the PEKK model in relation to tooth structure, cement line between the endocrown system and the tooth, and flowable composite as the following: (93.1, 64.5, 58.4 MPa) respectively, while Zirconia revealed lower maximum stress values (11.4, 13.6, 11.6 MPa) respectively. It means that, PEEK material presented maximum stress values than other tested materials. When these results compared with the individual enamel tensile strength (11.50 MPa), the PEKK stress value (93.1 MPa) exceeded it significantly followed by Vita-Enamic (24.2 MPa), while Nacera and Zirconia stress values (16.3, 11.4 MPa) respectively were nearly similar to it. The mvM stress levels for all materials did not reach the individual dentin tensile strength (98.70 MPa), particularly for PEKK, where its value was almost close to dentin tensile strength (Figs. 4, 5 and 6).

**Table 7:** Maximum modified von Mises stress (mvM) values (MPa) for tooth, cement lines, flowable composite for tested restorative materials

Structure (Material/Tissue)	Maximum modified von Mises stress (mvM)			
	**Vita-Enamic**	**Nacera Hybrid**	**Zirconia**	**PEKK**
**Tooth**	24.2	16.3	11.4	93.1
**Cement line**	29.6	20.4	13.6	64.5
**Flowable composite**	15.4	13.3	11.6	58.4

### Stress distribution in endocrown materials

**Fig. 7:**
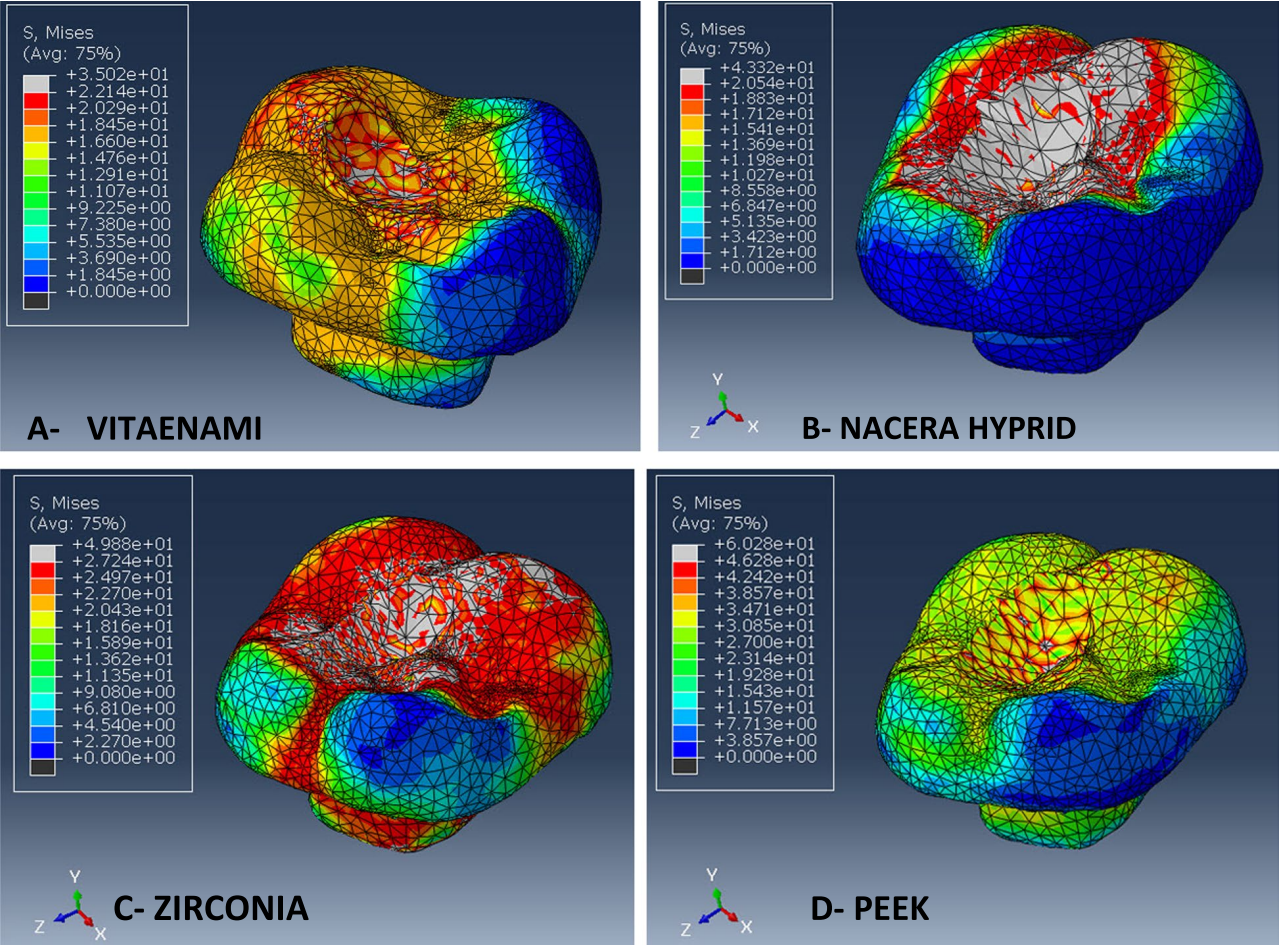
Showing stress distribution in tested endocrown materials

According to mvM analysis, PEKK restorative endocrown material displayed the greatest maximum stress distribution value (60.2 MPa), which is equal to 1625.4N. This result is consistent with the material’s fracture resistance rating (1845N). However, the Vita-Enamic restorative endocrown material displayed the lowest maximum stress value (35 MPa), which is equivalent to 945N. This finding is also consistent with the Vita-Enamic material's fracture resistance value (946N). The occlusal loading areas had the highest stress concentrations when the stress distribution pattern was properly considered, as seen by the colorimetric locations (red and light grey) (Table 8, Fig. 7).

**Table 8:** Stress distribution (MPa), Fracture force (N), and Fracture resistance (N) values of endocrown materials

Material	Stress distribution (MPa),	Fracture force (N)	Fracture resistance (N)
Vita-Enamic	35	945	946.5
Nacera-hybrid	43.3	1169	1135.8
Zirconia	49.8	1345	1367.2
PEKK	60.2	1625.4	1845.2

## Discussion

Through the use of 3D Finite Element analysis, the present in-vitro study was conducted to assess and compare the effects of different computer-aided design/manufacturing (CAD/CAM) materials on marginal adaption and fracture resistance of Endocrown restorations. Results of this in-vitro study demonstrated that there was statistically significant difference between different selected materials regarding to internal adaptation, marginal gap and fracture resistance, so the proposed null hypothesis that different CAD/CAM materials have no effect on the marginal integrity, and fracture resistance of Endocrown restoration with 3D Finite Element analysis was rejected.

Severely-damaged endodontically treated teeth (ETT) rehabilitation proceeds to be a challenging issue in dental practice. These teeth are usually restored using the conventional post-retained restorations. Recently, CAD/CAM technology with development of restorative materials and adhesive methods has made the conservative Endocrowns constitute a reliable promising restorative approach [46]. Different CAD/CAM materials have been designed as an alternate intra-radicular post-core material [32]. The clinical relevance of coronal restoration is critical to the long-term performance of ETT, not only in terms of restoring function but also in terms of protecting the remaining tooth structure and maintaining a good marginal quality [1]. In this in-vitro study, Endocrown was selected as a line of treatment because it is a minimally invasive procedure, so its advantages areprotection of established tooth structure, and does not need additional tooth structure removal, as there is no way to avoid it in post and core restoration. Extracted human molars were used in this study rather than metal, plastic, or bovine models because natural teeth simulate the modulus of elasticity, thermal conductivity, bonding properties, and strength of clinical situation [50].

All selected molars were vertically inserted in the center of a plastic ring filled with an epoxy resin material using a special centralizing device to ensure uniformity of location. In this research, self-cured epoxy resin was used because it has a modulus of elasticity (12GPa) comparable to that of human bone (18GPa), simulating the teeth in the alveolar bone. Whenever PDL layer is established around roots, it may act as a shock absorber that enables accurate tooth movement simulation with even stress distribution in the artificial PDL material [41]. Resembling other in-vitro studies [41, 51], and to simulate the supporting human bone, a rigid acrylic resin material with a nearly same modulus of elasticity was used to build a homogeneous 0.3 mm layer for PDL simulation around both roots. Additionally, a 2 mm layer of flowable composite was added to the pulp chamber floor above the gutta-percha level to seal the canal entrance and give a flat, uniform base [34].

The standardization in the present study was achieved by many methods. The first way was in the selection of molars as uniform dimension as possible in an effort to reduce confounding variability [8]. The second way was in the teeth preparation as it was performed by computerized numerical control milling machine (CNC) to prepare molars in standardized dimensions [11]. The butt margin design used in this study offers a configuration without thin or complex ferrule margins, reducing milling bur limitations in recreating the intaglio surface of endocrowns and allowing easy resin cement escape, resulting in proper seating and internal fit of all endocrowns with limited marginal gaps [52].

In present study, the cement space that used was 50 µm to ensure a good marginal seal and to allow the restoration to set more accurately [11, 34]. The space provided for the cementing agent has a direct effect on differences in marginal discrepancy. The choice of cement spacing less than 40 µm prevents the crown from setting, resulting in increased marginal discrepancy [53]. This study utilized the silicon replica technique (SRT), which is less expensive, simple to use, precise, and repeatable rapidly without loss of precision. It is also a non-destructive technique that does not cause damage to the abutment tooth or the restoration. However, this technique has disadvantage such as: 2- dimensional-based method, and there is a possibility of tearing and deformation of the impression materials. Some previous studies reported that, the silicone replica technique recorded higher reliability than the other methods [43, 54].

Translucent Prettau Zirconia, Vita-Enamic, Nacera Hybrid and Pekkton ivory materials were selected in this study. Regarding to clinical relevance Zirconia material is characterized by positive properties like a high flexural strength of up to 1,200 MPa, a high temperature resistance, as well as a constant shrinking factor granting the highest possible precision. Dental manufacturers tried to satisfy the interest for higher esthetic monolithic zirconia ceramics by developing special formulas of this restorative material. Because of multiple advantages of this material as its high flexural strength, good esthetics, and translucency, zirconia is frequently used in the construction of restorations. As a result, new translucent variations of zirconia have been created with superior optical qualities [55]. The second material selected in this study is Vita-Enamic material as it is the type of CAD/CAM material created to combine the benefits of ceramic and composite materials and known as polymer infiltrated ceramic material (PICM). According to the material composition, plasticity feature to the bulk material obtained due to the presence of both polymer and ceramic phases together within used (PICM) [11]. More favorable advantages have been reported for Vita-Enamic material such as the reasonable index of brittleness that allows it to be a suitable CAD/CAM material. Also it can be manipulated in one step without requiring additional firing such as some partially sintered CAD/CAM materials, this result in final products with a higher degree of dimensional accuracy. Comparing with traditional veneering porcelains, the lower material hardness provide better protection of opposing teeth against excessive wear as well as more rapid machining in CAD/CAM milling machines [11]. Another new CAD/CAM hybrid ceramic is the Nacera Hybrid material for permanent restorations and contains 50% Nano-glass and 50% polymer-matrix. This hybrid ceramic material has been introduced for manufacturing partial crowns, veneers and up to 3 units’ bridges (https://c4d.solutions/wp-content/uploads/2019/02/dmchybrid-anleitung-webseite_en-1.pdf).

A PEKK-based polymer (Pekkton ivory), material, is characterized as an attractive novel material for endocrown systems. Its application in this study for endocrowns was based on the fact that it is biocompatible and has mechanical qualities similar to those of normal teeth, which improves the biomechanical fit between the restoration and tooth and decreases the risk of fracture [11, 41]. It has a nearly similar compressive strength (246 MPa) to that of dentin (297 MPa), and also modulus of elasticity (5.1 GPa) nearly similar to dentin (18.6 GPa) and bone [34, 56]. Another reason for selecting PEKK in this study as its biological requirements are not a concern, since PEKK is an inert, non-allergenic polymeric biomaterial that has been suggested as an alternative to metal alloys in many types of prostheses [57].

The influence of various CAD/CAM materials on the marginal integrity, internal adaptability, and fracture resistance of endocrown restorations was studied in the current study. There was a significant difference between the four tested materials, according to the results of the internal and marginal fit based on the materials used (*P* < 0.05). Zirconia group showed the highest mean values of internal and marginal discrepancies (μm) among studied groups, while the lowest mean value of internal discrepancy (μm) was observed for Nacera group and the lowest mean value of marginal discrepancy (μm) was observed for PEKK group, as a result, the study’s null hypothesis was rejected.

The different physical properties of these materials, such as their hardness and their various fire shrinkage rates, may be responsible for the significant differences in internal and marginal discrepancy and fracture resistance between Zirconia and the other examined groups [58]. Additionally, a material's machinability in the milling system may change depending on its hardness, according to research by El Ghoul et al. (2020) [43], and other studies, who found an inverse relationship between a material’s hardness and machinability [59, 60].

The findings of this research demonstrated that, Endocrowns fabricated with zirconia recorded the highest mean values of internal and marginal discrepancies while PEKK presented the lowest mean values of marginal discrepancy than the other tested materials, also showed the lowest mean values of internal discrepancy compared to the other tested groups except Nacera hybrid group. Since these marginal and internal discrepancies can be regarded as being a part of the Endocrown restorations’ overall accuracy, these outcomes may be the result of a difference in the sintering process, which was thought to have an impact on fit as the zirconia group underwent sintering in the last step [61].updated While the PEKK group did not undergo sintering, the zirconia group did, and the greater discrepancy shown in the zirconia group was thought to be an error caused by inaccurately predicting shrinkage that occurred during the sintering process. These results were in agreement with those of Bae et al.., (2017) [62].

In this in-vitro study, marginal gap measurements showed no significant differences between Vita-Enamic and Nacera hybrid (86.55, 82.37 μm respectively), while there was significant difference between both materials regarding to internal discrepancy and fracture resistance as NH material showed lower mean values of internal discrepancy (69.27 μm) and showed higher mean values of fracture resistance (1135.80N) than VE material (83.15 μm, 946.50 N respectively). Also, mode of failure of both materials was close to each other. These results can be explained as both NH and VE materials are hybrid ceramics and have different microstructures. The moduli of elasticity of NH and VE materials are 9.9 GPa and 30.0 GPa respectively according to the manufacturer’s information. The composition of the NH is a ceramic material matrix consists of 50% Nano-glass and 50% polymer matrix, according to the manufacturer’s information, 100% silanized glass is permanently integrated into the polymer matrix [63]. Based on a ceramic network material with a polymer infiltration, Vita-Enamic has a dominating network (86wt. %) that is strengthened by an acrylic polymer network (14%). The two networks penetrate each other completely [64].updated This could possibly be attributed to the different compositions of these two hybrid ceramics with different filler contents, which have an impact on the much higher fracture resistance values and responsible for this significant differences in results.

On the other hand there was significant difference between all tested groups regarding to fracture resistance (*P* = 0.000), as the PEKK endocrowns showed the highest mean values of fracture resistance and the higher percentage of unfavorable fracture (70%). These results may be attributed to similar mechanical properties (compressive strength, modulus of elasticity and resilience) of this polymer material (PEKK) to that of natural dentition which enhance the reliability of the restorative system via producing a better biomechanical match between tooth and restoration [11, 41]. The compressive strength of PEKK material and tooth dentin is 246 MPa and 297 MPa, respectively, while the elastic modulus for both is 5.1 GPa for PEKK and 18.6 GPa for dentin [34, 56]. One of the advantages of this study is that PEKK group specimens showed higher values of fracture resistance which may be related to the precise manufacturing of PEKK material with better marginal adaptation and internal fit when compared with other tested materials. The increased compression strength and improved shock absorption of PEKK resulted in a lower stress concentration on the manufactured prosthesis, according to other research by Villefort et al. in (2022) [65]. The results of FEA are compatible with the results of practical part of this in-vitro study.

Analysis of the failure mode of the endocrowns restored teeth was as significant as considering the absence of fracture. It was evaluated to determine whether the remaining structure can be repaired after recording technical failure in clinical practice or not [66].

### Limitations of study

The current study’s limitations include not simulating the forces dynamically, such as during chewing cycles, and not simulating the saliva-filled intra-oral circumstances. These restrictions may be solved in future research, or an in vivo study may be done to examine the clinical effectiveness of endocrown restorations made from a variety of CAD/CAM materials and prepared at various depths. Also A polyvinyl siloxane impression material with a low viscosity was used in the replica technique in the current study. Lower viscosities might have different outcomes. Further future studies are thus needed to determine how different replica material consistencies affect internal adaptation and the marginal gaps when more ageing cycles are achieved using various techniques.

## Conclusions

Under the circumstances of this in-vitro analysis, it was determined that, with the exception of Nacera hybrid material (regard to internal discripancy); PEKK material demonstrated statistically excellent marginal and internal fit. Additionally the PEKK material explained the highest fracture resistance value which leads to increased demand for its use in the future in the dental world.

## Supplementary Information

**Additional file 1.** Raw data for marginal adaptation test of Vita Enamic Endocrowns.

**Additional file 2.** Raw data for marginal adaptation test of Nacera Hyprid Endocrowns.

**Additional file 3.** Raw data for marginal adaptation test of Zirconia Endocrowns.

**Additional file 4.** Raw data for marginal adaptation test of PEKK Endocrowns.

**Additional file 5.** Raw Data of Fracture resistance test of Endocrowns.

## Data Availability

The datasets used and/or analysed during the current study available from the corresponding author on reasonable request.
