# Supplementary material for: Impact of different CAD/CAM materials on internal and marginal adaptations and fracture resistance of endocrown restorations with: 3D finite element analysis
Source: BMC Oral Health. 2023 Jun 25;23:421. doi: 10.1186/s12903-023-03114-8 (PMC10291793; doi:10.1186/s12903-023-03114-8)
Supplement: Supplementary file 1 — Additional file 1. Raw data for marginal adaptation test of Vita Enamic Endocrowns. [file 12903_2023_3114_MOESM1_ESM.docx]

| Region | surface | E1 | E2 | E3 | E4 | E5 | E6 | E7 | E8 | E9 | E10 |
| --- | --- | --- | --- | --- | --- | --- | --- | --- | --- | --- | --- |
| Marginal  area | **Mesial** | 123 | 100 | 70 | 90 | 70 | 80 | 70 | 80 | 90 | 90 |
|  | **Distal** | 123 | 90 | 70 | 100 | 80 | 70 | 80 | 100 | 70 | 90 |
|  | **Buccal** | 133 | 70 | 90 | 100 | 70 | 70 | 80 | 90 | 100 | 70 |
|  | **Palatal** | 113 | 80 | 100 | 70 | 70 | 90 | 80 | 100 | 70 | 80 |
| Pulpal  Wall | **Mesial** | 64 | 70 | 80 | 60 | 80 | 70 | 60 | 70 | 70 | 80 |
|  | **Distal** | 115 | 100 | 70 | 80 | 70 | 70 | 80 | 80 | 100 | 70 |
|  | **Buccal** | 115 | 80 | 80 | 70 | 100 | 70 | 80 | 90 | 90 | 70 |
|  | **Palatal** | 64 | 70 | 100 | 80 | 90 | 70 | 90 | 100 | 70 | 80 |
| Pulpal  floor | **Mesial** | 95 | 90 | 80 | 100 | 70 | 80 | 100 | 90 | 80 | 80 |
|  | **Distal** | 112 | 100 | 90 | 70 | 80 | 80 | 90 | 70 | 100 | 80 |
|  | **Buccal** | 103 | 80 | 90 | 80 | 80 | 70 | 90 | 80 | 70 | 100 |
|  | **Palatal** | 104 | 90 | 100 | 70 | 90 | 100 | 70 | 80 | 90 | 80 |

**Raw data for marginal adaptation test of Vita Enamic Endocrowns**
