# Supplementary material for: Impact of different CAD/CAM materials on internal and marginal adaptations and fracture resistance of endocrown restorations with: 3D finite element analysis
Source: BMC Oral Health. 2023 Jun 25;23:421. doi: 10.1186/s12903-023-03114-8 (PMC10291793; doi:10.1186/s12903-023-03114-8)
Supplement: Supplementary file 2 — Additional file 2. Raw data for marginal adaptation test of Nacera Hyprid Endocrowns. [file 12903_2023_3114_MOESM2_ESM.docx]

**Raw data for marginal adaptation test of Nacera Hyprid Endocrowns**

| Region | surface | N1 | N2 | N3 | N4 | N5 | N6 | N7 | N8 | N9 | N10 |
| --- | --- | --- | --- | --- | --- | --- | --- | --- | --- | --- | --- |
| Marginal  area | **Mesial** | 97 | 90 | 90 | 80 | 70 | 90 | 80 | 70 | 90 | 90 |
|  | **Distal** | 91 | 90 | 80 | 70 | 80 | 70 | 80 | 80 | 90 | 90 |
|  | **Buccal** | 89 | 80 | 90 | 70 | 80 | 70 | 90 | 90 | 70 | 80 |
|  | **Palatal** | 98 | 90 | 80 | 70 | 80 | 90 | 70 | 80 | 90 | 70 |
| Pulpal  Wall | **Mesial** | 60 | 70 | 70 | 80 | 60 | 60 | 70 | 80 | 70 | 70 |
|  | **Distal** | 61 | 60 | 60 | 70 | 80 | 80 | 60 | 70 | 80 | 70 |
|  | **Buccal** | 57 | 60 | 70 | 80 | 70 | 60 | 70 | 70 | 70 | 80 |
|  | **Palatal** | 63 | 60 | 70 | 80 | 70 | 70 | 60 | 60 | 80 | 80 |
| Pulpal  floor | **Mesial** | 73 | 70 | 60 | 60 | 80 | 70 | 70 | 60 | 70 | 70 |
|  | **Distal** | 63 | 70 | 70 | 80 | 70 | 60 | 60 | 70 | 80 | 80 |
|  | **Buccal** | 66 | 70 | 60 | 70 | 70 | 80 | 80 | 60 | 70 | 70 |
|  | **Palatal** | 69 | 70 | 70 | 60 | 60 | 70 | 80 | 70 | 70 | 80 |
