# Supplementary material for: Impact of different CAD/CAM materials on internal and marginal adaptations and fracture resistance of endocrown restorations with: 3D finite element analysis
Source: BMC Oral Health. 2023 Jun 25;23:421. doi: 10.1186/s12903-023-03114-8 (PMC10291793; doi:10.1186/s12903-023-03114-8)
Supplement: Supplementary file 3 — Additional file 3. Raw data for marginal adaptation test of Zirconia Endocrowns. [file 12903_2023_3114_MOESM3_ESM.docx]

**Raw data for marginal adaptation test of Zirconia Endocrowns**

| Region | surface | Z1 | Z2 | Z3 | Z4 | Z5 | Z6 | Z7 | Z8 | Z9 | Z10 |
| --- | --- | --- | --- | --- | --- | --- | --- | --- | --- | --- | --- |
| Marginal  area | **Mesial** | 80 | 80 | 90 | 100 | 100 | 110 | 100 | 100 | 90 | 100 |
|  | **Distal** | 123 | 120 | 110 | 100 | 120 | 120 | 100 | 100 | 110 | 110 |
|  | **Buccal** | 113 | 110 | 120 | 100 | 110 | 100 | 110 | 120 | 100 | 100 |
|  | **Palatal** | 90 | 80 | 100 | 100 | 110 | 100 | 90 | 100 | 100 | 90 |
| Pulpal  Wall | **Mesial** | 90 | 110 | 120 | 100 | 90 | 90 | 100 | 100 | 90 | 110 |
|  | **Distal** | 71 | 70 | 90 | 100 | 80 | 80 | 90 | 100 | 90 | 100 |
|  | **Buccal** | 76 | 70 | 80 | 100 | 80 | 90 | 100 | 100 | 90 | 70 |
|  | **Palatal** | 85 | 80 | 100 | 100 | 90 | 80 | 80 | 90 | 100 | 90 |
| Pulpal  floor | **Mesial** | 115 | 110 | 120 | 120 | 100 | 110 | 100 | 120 | 100 | 110 |
|  | **Distal** | 114 | 110 | 120 | 100 | 100 | 110 | 110 | 120 | 100 | 110 |
|  | **Buccal** | 109 | 110 | 120 | 100 | 110 | 100 | 110 | 110 | 120 | 100 |
|  | **Palatal** | 114 | 110 | 100 | 100 | 110 | 120 | 120 | 110 | 110 | 120 |
