# Supplementary material for: Impact of different CAD/CAM materials on internal and marginal adaptations and fracture resistance of endocrown restorations with: 3D finite element analysis
Source: BMC Oral Health. 2023 Jun 25;23:421. doi: 10.1186/s12903-023-03114-8 (PMC10291793; doi:10.1186/s12903-023-03114-8)
Supplement: Supplementary file 4 — Additional file 4. Raw data for marginal adaptation test of PEKK Endocrowns. [file 12903_2023_3114_MOESM4_ESM.docx]

**Raw data for marginal adaptation test of PEKK Endocrowns**

| Region | surface | P1 | P2 | P3 | P4 | P5 | P6 | P7 | P8 | P9 | P10 |
| --- | --- | --- | --- | --- | --- | --- | --- | --- | --- | --- | --- |
| Marginal  area | **Mesial** | 69 | 70 | 70 | 60 | 80 | 100 | 70 | 90 | 60 | 70 |
|  | **Distal** | 100 | 80 | 70 | 90 | 80 | 100 | 70 | 90 | 80 | 70 |
|  | **Buccal** | 80 | 70 | 90 | 80 | 80 | 90 | 70 | 80 | 70 | 90 |
|  | **Palatal** | 70 | 80 | 80 | 90 | 70 | 70 | 80 | 70 | 80 | 80 |
| Pulpal  Wall | **Mesial** | 80 | 90 | 100 | 90 | 80 | 70 | 70 | 80 | 100 | 90 |
|  | **Distal** | 60 | 70 | 70 | 60 | 80 | 60 | 70 | 80 | 90 | 80 |
|  | **Buccal** | 70 | 80 | 100 | 90 | 90 | 70 | 80 | 70 | 90 | 100 |
|  | **Palatal** | 65 | 80 | 70 | 60 | 60 | 90 | 70 | 60 | 60 | 100 |
| Pulpal  floor | **Mesial** | 90 | 100 | 90 | 80 | 90 | 100 | 70 | 70 | 80 | 80 |
|  | **Distal** | 80 | 70 | 90 | 100 | 100 | 70 | 80 | 90 | 70 | 70 |
|  | **Buccal** | 95 | 90 | 80 | 80 | 70 | 90 | 90 | 70 | 70 | 90 |
|  | **Palatal** | 85 | 80 | 80 | 100 | 90 | 90 | 70 | 80 | 80 | 70 |
