# Supplementary material for: Impact of different CAD/CAM materials on internal and marginal adaptations and fracture resistance of endocrown restorations with: 3D finite element analysis
Source: BMC Oral Health. 2023 Jun 25;23:421. doi: 10.1186/s12903-023-03114-8 (PMC10291793; doi:10.1186/s12903-023-03114-8)
Supplement: Supplementary file 5 — Additional file 5. Raw Data of Fracture resistance test of Endocrowns. [file 12903_2023_3114_MOESM5_ESM.docx]

**Raw Data of Fracture resistance test of Endocrowns (N)**

| N | E group | N group | Z group | P group |
| --- | --- | --- | --- | --- |
| 1 | 1241 | 1250 | 1331 | 2483 |
| 2 | 1078 | 980 | 2250 | 1738 |
| 3 | 1154 | 1329 | 915 | 1831 |
| 4 | 1132 | 1328 | 1240 | 2381 |
| 5 | 2220 | 1127 | 1170 | 1876 |
| 6 | 980 | 1865 | 2259 | 2431 |
| 7 | 1324 | 1267 | 1076 | 4215 |
| 8 | 1231 | 990 | 920 | 3420 |
| 9 | 1165 | 1798 | 1324 | 2376 |
| 10 | 1098 | 1434 | 1543 | 1987 |
